# Cilioretinal Artery Occlusion Combined with Central Retinal Vein Occlusion: What Is the Best Imaging Modality for the Follow-Up?

**DOI:** 10.1155/2017/9032576

**Published:** 2017-12-21

**Authors:** Mahmut Kaya, Taylan Ozturk, Ziya Ayhan, Nilufer Kocak, Suleyman Kaynak

**Affiliations:** Department of Ophthalmology, Dokuz Eylul University School of Medicine, Izmir, Turkey

## Abstract

We report retinal structural changes of a 37-year-old man diagnosed with the concomitant occlusion of cilioretinal artery and central retinal vein. Comprehensive ophthalmological evaluation was performed, followed by spectral-domain optical coherence tomography (SD-OCT, Heidelberg), optical coherence tomography angiography (OCT angiography, Optovue Inc., Fremont, California, USA), fluorescein angiography, and color fundus photography. The use of OCT angiography and en face SD-OCT imaging as an adjunct test to map out correlative paracentral scotomas during follow-up allowed us to evaluate cilioretinal artery occlusion in the best way due to obtaining satisfactory images of the normal retinal vascular networks and areas of nonperfusion and congestion at various retinal levels.

## 1. Introduction

The cilioretinal artery occlusion is very rare and accounts for 5% of retinal artery occlusions [1]. Optical coherence tomography angiography (OCT angiography) is a new imaging technology that allows for fast, noninvasive assessment of microvascular perfusion across the macular region, offering the potential to perform quantitative assessment. OCT angiography has the capability to segment each layer of the retinal microvasculature in normal and pathological eyes without dye injection [2]. Herein, we aimed to characterize the appearance of the peri- and parafoveal macular microvasculature in a visually asymptomatic young patient with the cilioretinal artery occlusion in chronic phase using OCT-A and to compare different imaging modalities.

## 2. Case Report

A 37-year-old man without any history of ocular and systemic pathology presented with painless visual decrease in his right eye for 10 days. He had suffered from multiple episodes of amaurosis fugax for 30 days. Clinical examination, spectral-domain OCT (SD-OCT), OCT angiography (XR Avanti, software version 2015.1.1.98, Optovue Inc., Fremont, California, USA), fluorescein angiography, and color fundus photography, as well as systemic and laboratory assessments, were used to document findings in the patient with cilioretinal artery occlusion combined with central retinal vein occlusion. The patient presented in this report has given informed consent for this publication.

**Figure 1:**
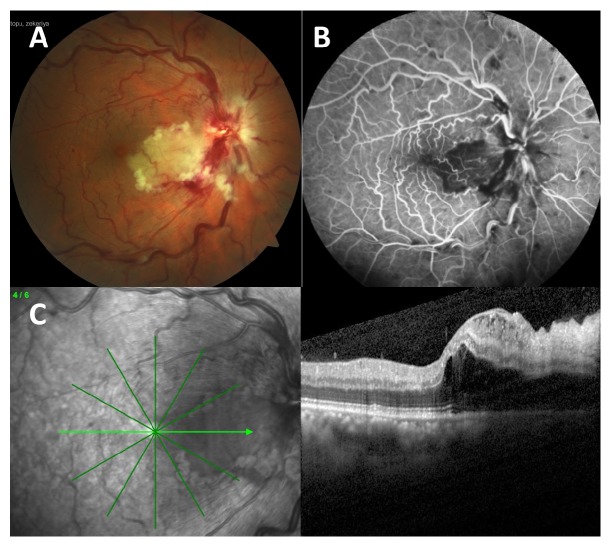
Color fundus photographs of the right eye (A) at the time of presentation. On fluorescein angiography (B), nonperfusion of the cilioretinal artery was shown in the area of cilioretinal at the initial presentation. Optical coherence tomography (OCT) at baseline (C) showed the presence of intraretinal and subretinal fluid and hyperreflective inner retinal layers.

At the first visit, his best-corrected visual acuity (BCVA) was 20/60 in the right eye (RE) and 20/20 in the left eye (LE). Fundus examination of the RE demonstrated a whitening of the retina along the distribution of the cilioretinal artery. The retinal veins were mildly dilated and tortuous and accompanied by adjacent retinal haemorrhages (Figure 1(A)). Fluorescein angiography demonstrated delayed filling and emptying of this artery (Figure 1(B)). A SD-OCT examination revealed retinal hyperreflectivity and thickening with loss of distinction of retinal layers (Figure 1(C)). Based on these findings, the patient was ultimately diagnosed with a combined cilioretinal artery and central retinal vein occlusion. His blood laboratory findings, systemic physical examination, electrocardiogram, carotid ultrasound imaging, and chest X-ray were all unremarkable. Echocardiography revealed normal findings. Cryoglobulin, lupus anticoagulant, and anti-cardiolipin antibodies were all negative. Antithrombin III, protein C, and protein S activities were normal. Dexamethasone intravitreal implant (Ozurdex, Allergan Inc., Irvine, California, USA), which is the first-line treatment option according to health insurance policy in Turkey, was injected into the right vitreous cavity to at least alleviate the central retinal vein occlusion-related concomitant optic disc and macular edema. The patient was examined monthly over a 6-month period.

**Figure 2:**
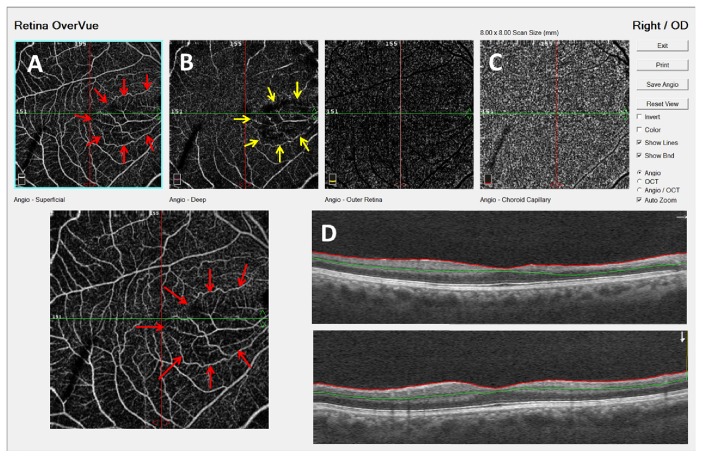
OCT angiography was performed 10 months later. Wedge-shaped area of capillary nonperfusion was revealed in both the superficial (A) and deep (B) retinal capillary plexus in area supplied by the cilioretinal artery and the choriocapillaris (C) was not affected.

**Figure 3:**
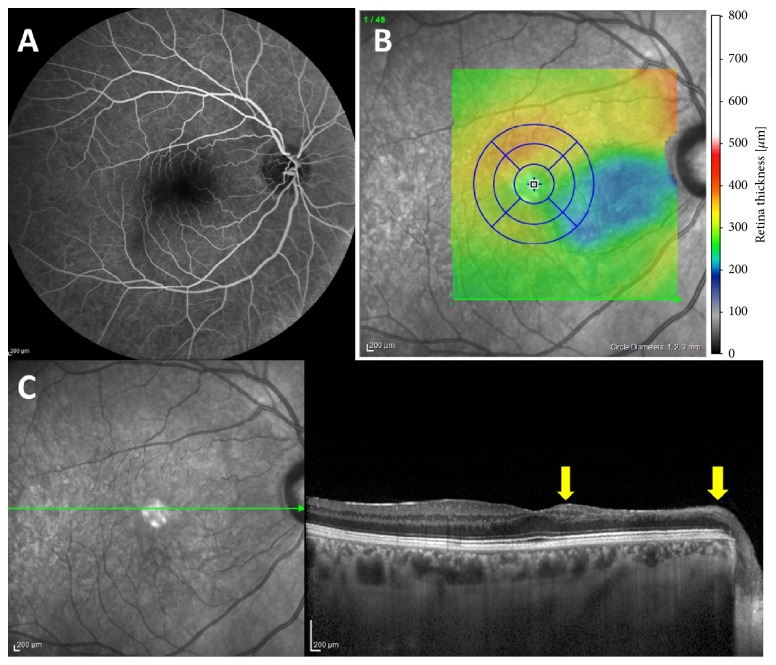
At follow-up, early phase of FA showing quite normal filling of the cilioretinal artery sparing (A). The retinal capillary network is well perfused on this magnification of the posterior pole. Spectral-domain OCT density map delineating areas of atrophy (blue) corresponding to cilioretinal arteriole occlusions (B). Spectral-domain OCT delineating areas of atrophy (arrows) corresponding to distribution of cilioretinal artery occlusion (C).

Upon follow-up, BCVA had increased to 20/20, the haemorrhages were absorbed, and the dilatation and tortuosity of retinal vessels had resolved. In OCT angiography, of the affected RE, there was attenuation of both the superficial capillary plexus and deep capillary plexus (Figure 2). The circulation of the retinal vessels had improved on FA (Figure 3(A)). The SD-OCT density map clearly delineates the areas of atrophy corresponding to the distribution of the sclerotic arterioles (Figure 3(B)). Spectral-domain OCT scan demonstrated diffuse thinning of the inner nuclear layer, corresponding to the central zone of the cilioretinal artery occlusion (Figure 3(C)). Color fundus photography was unremarkable.

## 3. Discussion

The superficial retinal capillary plexus is located in the nerve fiber layer near the disc, but is present more predominantly within the ganglion cell layer in the central macular region [3–5]. The deep retinal capillary plexus is composed of an intermediate and deep plexus located at the inner and outer planes of the inner nuclear layer [3–5]. These capillary layers are interconnected by perpendicularly oriented vessels [6] and may be disproportionately affected by ischemic retinal vascular disease. Although retinal vasculature changes in retinal artery occlusions are widely described in the literature, precise assessment and analysis of the deep retinal capillary plexus with FA is limited mostly by light scattering from the inner retinal layers. At the follow-up, not all imaging modalities are useful for retinal artery occlusion, so, for retina specialists, it is important to be well informed in order to make the best choice from all of the various available imaging modalities [4, 5].

In this case in the chronic phase, on OCT angiography, we demonstrated nonperfusion of both the superficial and deep capillary plexus levels in areas with persistent ischemia. In chronic cilioretinal artery occlusion, regression of retinal edema is usually followed by development of retinal thinning and subsequent disorganization of the inner retinal architecture. En face OCT showed delineating areas of atrophy corresponding to distribution of cilioretinal arterial occlusion. However, FA did not show measurable filling delay and the capillary network seemed normally perfused.

When evaluating the acute phase of the isolated retinal artery occlusion, FA technique is an important diagnostic tool to reveal the affected arterial vasculature, with typically normal choroidal filling. However, it is limited to imaging only the superficial vascular plexus. Fluorescein angiogram provides information regarding the localization and extent of vascular disease. In chronic phase, FA did not consistently reveal any correlation to these lesions.

In the acute phase, OCT images demonstrate the increased reflectivity and thickness of the inner retina and a corresponding decrease of reflectivity in the outer layer of the retina and retinal pigment epithelium/choriocapillaris layer. Follow-up OCT images demonstrate a decrease in the reflectivity and thickness of the inner retinal layers and a corresponding increase of reflectivity in the outer retina and retinal pigment epithelium/choriocapillaris layer compared with the baseline OCT image, suggesting a generalized atrophy of the neurosensory retina as a late finding [3]. Therefore, the use of OCT may help facilitate prompt recognition of acute and chronic cilioretinal artery occlusion. En face OCTA may prove useful to quantify and further localize the foci of retinal ischemia in retinal artery occlusive disorders as demonstrated by SD-OCT [5].

OCT angiography represents a relatively new technology with the ability to not only noninvasively image the superficial capillary plexus traditionally seen on fluorescein angiography but also capture the flow of the deeper capillary plexuses. In the chronic phase, there were pruning and dropout of the deeper plexuses on OCT angiography matching the middle retinal atrophy [2, 5]. When compared to the current standard of FA, OCT angiography is fast and noninvasive and can provide improved visualization of the microvasculature.

The use of OCT angiography and en face SD-OCT imaging as an adjunct test to map out correlative paracentral scotomas during follow-up allowed us to evaluate cilioretinal artery occlusion in the best way due to obtaining satisfactory images of the normal retinal vascular networks and areas of nonperfusion and congestion at various retinal levels. Furthermore, OCT angiography might help to assess accurately the extent of macular ischemia and vascular flow changes during the course of retinal vascular occlusions.
